# Supplementary material for: Diagnostic Performance of Serum Erythropoietin to Discriminate Polycythemia Vera from Secondary Erythrocytosis through Established Subnormal Limits
Source: Diagnostics (Basel). 2024 Aug 29;14(17):1902. doi: 10.3390/diagnostics14171902 (PMC11393970; doi:10.3390/diagnostics14171902)
Supplement: Supplementary file 1 [file diagnostics-14-01902-s001.zip › 240825 Diagnostics supplementary table 1 2.pdf]

Supplemental Table S1. Spearman's rank correlation between serum erythropoietin and clinical and laboratory parameters in 393 healthy donors

	Rho	*P*-Value	
Hemoglobin	-0.233	<0.001	*
Hematocrit	-0.249	<0.001	*
White blood cells	-0.23	<0.001	*
Absolute neutrophil count	-0.208	<0.001	*
Platelets	-0.058	0.220	
HbA1c	-0.092	0.065	
Glucose	-0.075	0.117	
Age	-0.003	0.953	
Body mass index	-0.008	0.873	
Blood urea nitrogen	0.079	0.097	
Creatinine	-0.084	0.079	
Heart rate	0.002	0.963	
Diastolic blood pressure	-0.101	0.041	*
Systolic blood pressure	-0.075	0.129	
Total cholesterol	-0.047	0.330	
Triglyceride	-0.096	0.045	*
Low-density lipoprotein	-0.046	0.421	
High-density lipoprotein	0.224	0.007	*
Estimated glomerular filtration rate	0.033	0.494	

*$P < 0.05$

Supplemental Table S2. Comparison of clinical and laboratory parameters according to the functional limit of serum erythropoietin level (7.0 IU/L) in patients with secondary erythrocytosis and healthy donors

	subnormal EPO (< 7.0 IU/L) (n=85)	normal or increased EPO (≥ 7.0 IU/L) (n=357)	*P* - value	
Hemoglobin (g/dL)	15.3 (14.2 - 16.9)[1]	14 (13.2 - 15.3)	<0.0001	*
Hematocrit (%)	46.2 (42.2 - 49.9)	42.2 (39.6 - 45.5)	<0.0001	*
White blood cells ((10^9/L))	6.62 (5.45 - 8.43)	5.81 (4.80 - 7.00)	<0.0001	*
Absolute neutrophil count (10^9/L)	3.94 (2.94 - 5.46)	3.185 2.430 - 4.080	<0.0001	*
Platelets (10^9/L)	257 (234.8 - 304.8)	255 (222.0 - 290.0)	0.087	
HbA1c (%)	5.6 (5.4 - 5.7)	5.5 (5.3 - 5.7)	0.427	
Glucose (mg/dL)	99.0 (91.8 - 104.3)	96.0 (91.0 - 103.0)	0.270	
Age	49.0 (39.0 - 57.0)	52.0 (38.0 - 59.0)	0.288	
Body mass index	24.3 (22.3 - 26.9)	23.9 (22.0 - 26.7)	0.466	
Blood urea nitrogen (mg/dL)	12.6 (10.4 - 15.3)	13.4 (11.2 - 15.6)	0.192	
Creatinine (mg/dL)	0.80 (0.66 - 0.92)	0.73 (0.64 - 0.85)	0.011	*
Heart rate	75.0 (66.3 - 83.3)	75.0 (67.0 - 83.0)	0.889	
Diastolic blood pressure	79.0 (70.0 - 89.0)	77.0 (70.0 - 83.0)	0.028	*
Systolic blood pressure	126.5 (119.5 - 138.0)	123.0 (116.0 - 132.0)	0.008	*
Total cholesterol (mg/dL)	195.0 (170.0 - 223.5)	199.0 (171.0 - 222.0)	0.942	
Triglyceride (mg/dL)	123.0 (80.5 - 153.0)	99.0 (68.0 - 150.3)	0.095	
Low-density lipoprotein (mg/dL)	122.5 (97.5 - 146.5)	118.0 (96.0 - 139.0)	0.398	
High-density lipoprotein (mg/dL)	46.0 (38.0 - 53.0)	51.0 (44.5 - 60.0)	0.007	*
Estimated glomerular filtration rate (mL/min/1.73 ㎡)	103.0 (92.0 - 109.0)	100.0 (94.0 - 110.0)	0.837	
Hypertension	22.4 %	14.3 %	0.067	
Diabetes	3.5 %	3.4 %	0.939	
Obesity	10.8 %	5.9 %	0.107	

$*P < 0.05$
