# Supplementary material for: Diagnostic Performance of Serum Erythropoietin to Discriminate Polycythemia Vera from Secondary Erythrocytosis through Established Subnormal Limits
Source: Diagnostics (Basel). 2024 Aug 29;14(17):1902. doi: 10.3390/diagnostics14171902 (PMC11393970; doi:10.3390/diagnostics14171902)
Supplement: Supplementary file 1 [file diagnostics-14-01902-s001.zip › Supplemental Figure S1.pdf]

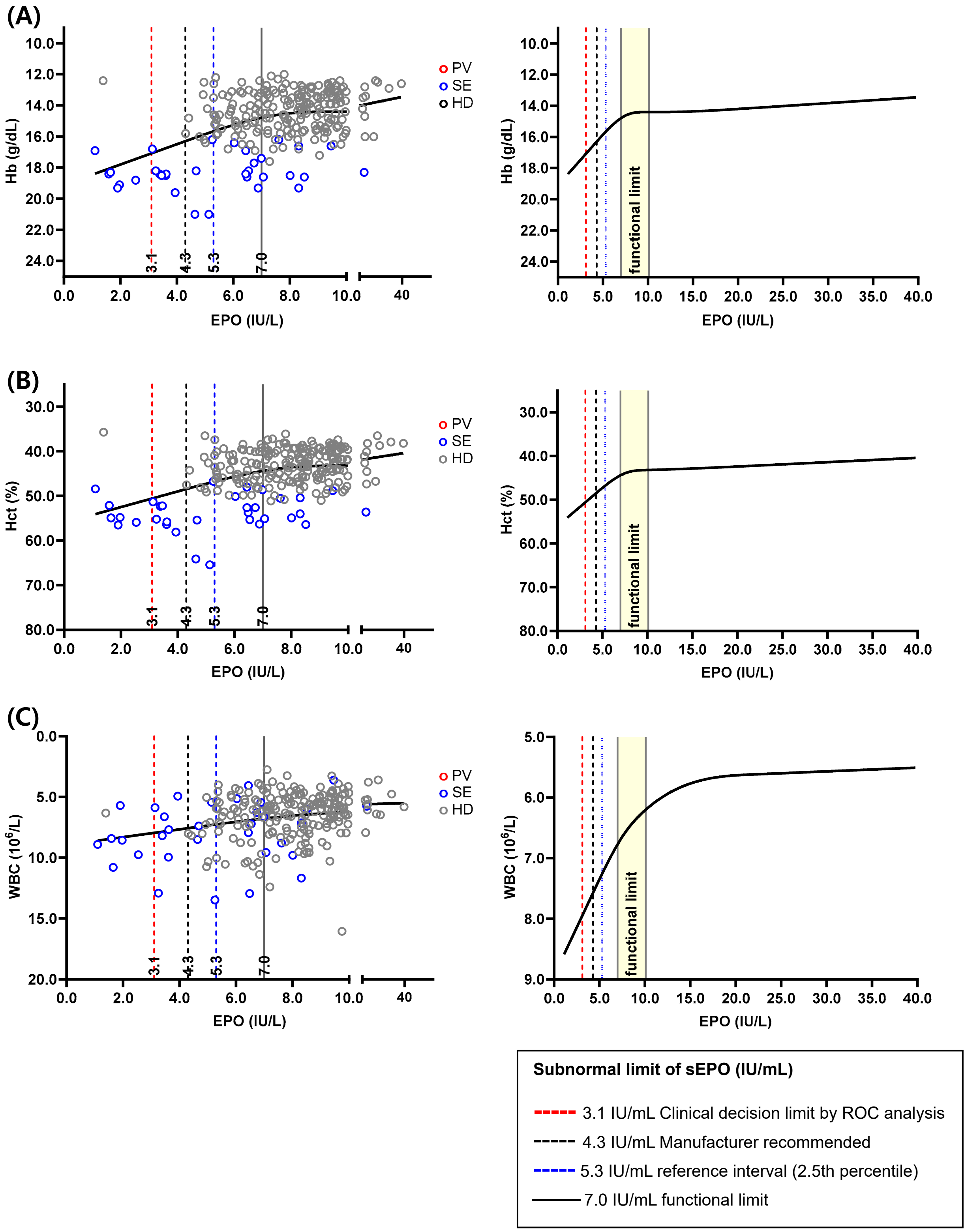

Supplementary Figure S1. Relations between serum erythropoietin (sEPO) and interrelated biomarkers in different population; (A) hemoglobin (Hb), (B) hematocrit (Hct), and (C) white blood cell (WBC). The sEPO and three biomarkers from 393 healthy donors (HDs) and 49 secondary erythrocythemia (SE) patients were correlated using plot smoothing splines model. The functional reference limit of sEPO, 7.0 IU/L, is represented by concentrations that show sharp changes in Hb and Hct values, but more modest changes in WBC. Established 'subnormal' limits of sEPO are shown as dashed vertical lines, and their values were smaller than the estimated functional reference limit.
